# Conversion from Venovenous to Venoarterial Extracorporeal Membrane Oxygenation in Adults

**DOI:** 10.3390/membranes11030188

**Published:** 2021-03-09

**Authors:** Lars Falk, Alexander Fletcher-Sandersjöö, Jan Hultman, Lars Mikael Broman

**Affiliations:** 1 ECMO Centre Karolinska, Department of Pediatric Perioperative Medicine and Intensive Care, Karolinska University Hospital, 17176 Stockholm, Sweden; jan.hultman45@gmail.com (J.H.); lars.broman@sll.se (L.M.B.); 2 Department of Physiology and Pharmacology, Karolinska Institutet, 171 76 Stockholm, Sweden; 3 Department of Clinical Neuroscience, Karolinska Institutet, 171 76 Stockholm, Sweden; alexander.fletcher-sandersjoo@sll.se; 4 Department of Neurosurgery, Karolinska University Hospital, 171 76 Stockholm, Sweden

**Keywords:** extracorporeal membrane oxygenation, conversion, venoarterial, venovenous, ECMO, VA, VV

## Abstract

No major study has been performed on the conversion from venovenous (VV) to venoarterial (VA) extracorporeal membrane oxygenation (ECMO) in adults. This single-center retrospective cohort study aimed to investigate the incidence, indication, and outcome in patients who converted from VV to VA ECMO. All adult patients (≥18 years) who commenced VV ECMO at our center between 2005 and 2018 were screened. Of 219 VV ECMO patients, 21% (*n* = 46) were converted to VA ECMO. The indications for conversion were right ventricular failure (RVF) (65%), cardiogenic shock (26%), and other (9%). In the converted patients, there was a significant increase in Sequential Organ Failure Assessment (SOFA) scores between admission 12 (9–13) and conversion 15 (13–17, *p* < 0.001). Compared to non-converted patients, converted patients also had a higher mortality rate (62% vs. 16%, *p* < 0.001) and a lower admission Respiratory Extracorporeal Membrane Oxygenation Survival Prediction (RESP) score (*p* < 0.001). Outcomes were especially unfavorable in those converted due to RVF. These results indicate that VA ECMO, as opposed to VV ECMO, should be considered as the first mode of choice in patients with respiratory failure and signs of circulatory impairment, especially in those with impaired RV function. For the remaining patients, Pre-admission RESP score, daily echocardiography, and SOFA score trajectories may help in the early identification of those where conversion from VV to VA ECMO is warranted. Multi-centric studies are warranted to validate these findings.

## 1. Introduction

Extracorporeal membrane oxygenation (ECMO) has been successfully employed in pediatric and neonatal patients since the 1980s [1] and is now being used more widely in adults as well [2,3,4,5]. There are three basic modes of ECMO: venovenous (VV), venoarterial (VA) and venovenoarterial (VVA) [6,7]. VV ECMO provides respiratory support, while VA and VVA ECMO are used when combined cardiorespiratory support is needed.

During treatment with VV ECMO, cardiocirculatory failure, including right ventricular failure (RVF), may develop and necessitate a need for circulatory support [8]. To achieve this, the workload of the right ventricle can be alleviated by reducing ventilator pressures, decreasing right ventricular (RV) afterload, removing fluid overload, and appropriate inotropic support. However, if these measures are insufficient, conversion to VA ECMO must be considered. The literature on ECMO mode conversions is sparse. One reason may be that before the Influenzae H1N1 pandemic in 2009, only a few centers offered ECMO for adults, and most patients were treated at cardiothoracic units dominated by VA ECMO. Thus, the awareness of RV and pulmonary interactions and monitoring, as well as experience in all ECMO modalities, permeated care. During and after the pandemic, adult respiratory ECMO has expanded into medical ICUs. Today, VV ECMO has become the dominating modality, and many ‘respiratory ECMO centers’ offer VV but not VA ECMO due to lack of experience and training. Consequently, conversion may not be considered in cases of RV failure leading to exacerbation of multiorgan failure and increased risk of death. Respiratory ECMO patients at risk include those with air-leak syndrome, acute respiratory distress syndrome, sepsis, fluid overload, and other diagnoses where lung rest is applied [9,10].

Conversion between ECMO modes was first described by Bartlett et al. in neonatal patients with progressive circulatory failure [11,12]. Despite being an accepted form of treatment for failing circulation in pediatric and neonatal VV ECMO patients today, conversions are poorly described in the literature. Evidence is especially scarce in adults. An ELSO Registry (Extracorporeal Life Support Organization, Ann Arbor, MI, USA) study of 64 neonates with diaphragmatic hernia, who were converted from VV to VA ECMO, reported no difference in ventilation strategies or circulation at the initiation of ECMO between those who were converted and not [13]. The indications for conversion, as well as data on the time of the decision to convert, were not presented. Another more recent ELSO Registry study of neonates and pediatric patients found that conversion from VV to VA ECMO was associated with increased mortality in both age groups [14]. In adults, Kon et al. reported a 4.1% conversion frequency in their study of 717 ECMO treatments from the ELSO Registry [15]. Data from the most recent ELSO Registry Report (Jan 2020) [2] showed that 1.4% of all VV ECMO patients from 2015 were converted from VV to VA ECMO, for whom the mortality was 73% [2]. Data from our unit has indicated a conversion rate of 16% in adult patients admitted for VV ECMO due to septic shock [16]. A similar frequency was found in our general ECMO cohort by von Bahr et al. [17]. Thus, data is scarce regarding both the incidence of and outcome following conversion in ECMO-treated adult patients.

The aim of this study was to review our institutional experience of patients who required conversion from VV to VA ECMO in order to provide population-based observational data on the indications for, the incidence of, and outcome following the procedure.

## 2. Materials and Methods

### 2.1. Patients

All adult (≥18 years) patients admitted for VV ECMO support at ECMO Centre Karolinska, between January 2005 and December 2018, were eligible for inclusion. Patients who were partially treated at a different hospital or who underwent cardiopulmonary resuscitation during cannulation were excluded. Patients converted to VA ECMO within the first six hours of ECMO treatment were also excluded. This time frame was chosen since (1) patients who have not stabilized on VV ECMO within a few hours often require VA ECMO support (i.e., to include the ‘wrong mode’ from the start would confound our study) and (2) a few uncertain cases may have been precautiously cannulated for VA ECMO, as 80% of our patients were cannulated at a referring hospital with a 3–6 h transport time ahead of them [18,19].

In accordance with the routine standard of care, all patients were treated with a positive end expiratory pressure (PEEP), low tidal volume (< 6 mL/kg ideal body weight), removal of fluid overload, pulmonary vasodilators, and optimized inotropic therapy. A tracheostomy was performed within the first 2–3 days, with the goal of keeping the patient awake and allowing pressure support ventilation. The typical patient may have higher tidal volumes in this phase of ECMO treatment than in many comparable European centers [20].

### 2.2. Variables

Data was retrospectively obtained from digital medical records and hospital charts. The following data were collected for all patients at ECMO initiation: sex, age, ECMO mode, Simplified Acute Physiology Score (SAPS-3) [21,22] and Respiratory Extracorporeal membrane oxygenation Survival Prediction (RESP) score [23], (http://www.respscore.com, accessed on 26 December 2020)—a score developed from ELSO Registry data and used to help compare ECMO cohorts.

The following additional data were collected for patients who were converted from VV to VA ECMO: body weight (on admission), ventilator settings, arterial blood gas data, blood pressure, number of vasoactive drugs, as well as vasoactive inotropic score [24] (VIS) (on admission, after 12 h of ECMO support and before conversion to VA). The Sequential Organ Failure Assessment (SOFA) score [25] was also calculated upon ECMO initiation (SOFA_in_) and at the time of conversion to VA ECMO (SOFA_conv_). We used the SOFA score to quantify organ failure severity and trajectory over time. Survival After Venoarterial ECMO (SAVE) [26] score was also calculated at the time of conversion (http://www.save-score.com, accessed on 26 December 2020).

Echocardiography, a well-accepted technique to assess central hemodynamics during ECMO [27,28,29], and RV function [10,20,30], was used to identify RVF and biventricular cardiac dysfunction leading to circulatory failure and circulatory shock. The time from diagnosis of RVF or circulatory shock to conversion was also noted. The circulatory shock was defined as a circulatory failure with a norepinephrine demand > 0.1 μg/kg per minute unresponsive to volume substitution (targeted mean arterial blood pressure 65 mmHg). Left ventricular heart failure (LVF) was defined as the clinically emergent onset of reduced cardiac output and reduced ejection fraction (<40%) assessed by echocardiography, leading to reduced S_PRE_O_2_ and/or increased plasma lactate. Right ventricular failure was defined as end-diastolic dilatation of the right ventricle, or a tricuspid annular plane systolic excursion of less than < 16 mm [30,31,32,33]. A D-shaped left ventricle during end-diastole without dilatation of the RV was not regarded as RVF, but rather suggestive of RV overload without failure unless the pulmonary flow was clearly decreased. Moreover, the levels of pulmonary flow and pulmonary pressures were taken into consideration. These data were extracted from echocardiography exams and the examiner’s documented findings of evidence for RVF.

### 2.3. Statistical Analysis

Data were tested for normal distribution using a Shapiro-Wilk test. Normally distributed data are presented as mean (± 1 SD), non-parametric data as median (25–75% quartiles), and categorical data as numbers (proportion). A *t-*test or ANOVA was used for comparison of normally distributed data, and a Mann-Whitney U test, Wilcoxon signed-rank test, or Kruskal-Wallis test was used for comparison of non-parametric data. For categorical variables, a Fisher’s exact test or chi-square test were used depending on sample size distribution. The statistical significance level was *p* < 0.05.

### 2.4. Ethical Considerations

The study was approved by the Regional Ethical Review Board in Stockholm, Sweden (DNR: 2017/1671-32), who waived the need for informed consent.

## 3. Results

**Figure 1:**
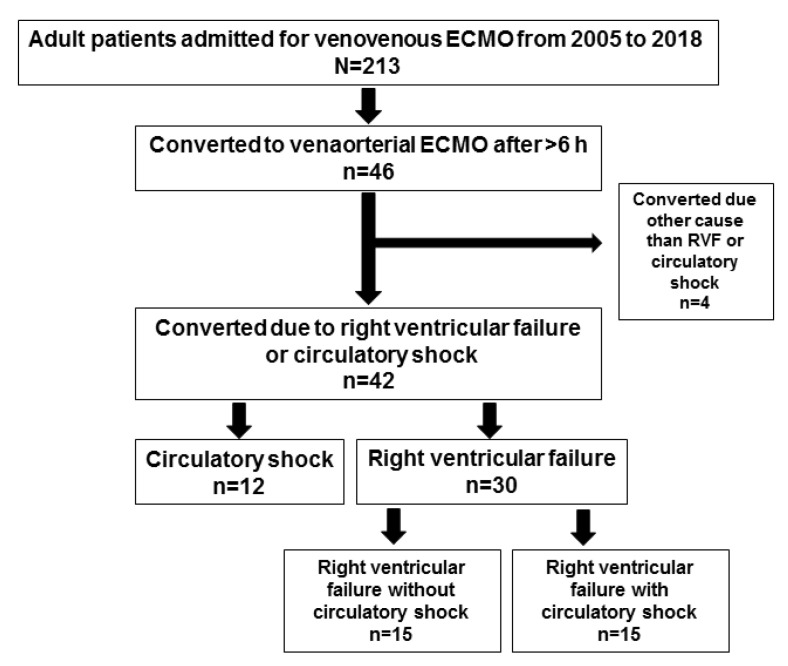
Consort diagram of the retrospective selection of patients. ECMO: extracorporeal membrane oxygenation; RVF: right ventricular failure.

In total, 213 adult VV ECMO patients were screened (Figure 1 and Table 1), of whom 46 (21%) were converted to VA ECMO (Supplemental Digital File Table S1).

The main causes for critical care were infectious in 72% (33/46) of the cases (bacterial pneumonia (*n* = 19); viral pneumonia (*n* = 6); sepsis/non-trauma acute respiratory distress syndrome (*n* = 8)), aspiration 9% (*n* = 4), trauma (*n* = 3), immune-related/cancer (*n* = 5), and one case of pulmonary edema. Patients who required conversion from VV to VA ECMO had significantly lower admission RESP scores compared to the non-converted cohort (−2.5 (−4–1) vs. 2 (0–4), *p* < 0.001). No such difference was observed for admission SAPS-3 scores (74.2 ± 11.6 vs. 74.1 ± 11.3, *p* = 0.99. During the first 12 h of VV support, arterial oxygen saturation (SO_2_) increased, pH normalized, and respiratory rate and tidal volume (Vt) were decreased while ventilator pressures and fraction inspired oxygen (FiO_2_) were adjusted to lower settings (Table 2). During the same time, the SOFAc (the circulatory domain of the SOFA score) decreased from 3 (3–4) to 3 (0–4) (*p* < 0.02) and VIS from 10 (3.8–38) to 4 (0–21) (*p* < 0.0001). Mean arterial blood pressure was maintained at 65 mmHg. At the time of conversion, positive end-expiratory pressure had been further reduced, Vt was reduced since the start of ECMO, and SO_2_ had dropped from 88 (79–93) to 80 (71–88) (*p* < 0.001) %. FiO_2_, SOFAc, the number of vasoactive drugs, and VIS were all significantly higher than during early ECMO support (Table 2). ECMO blood flow was the same at 12 h and at time of conversion, 4.3 (3.85–4.8) and 4.5 (4.1–5.0) L/min), respectively; mean arterial pressure (MAP) was unchanged. The median time from initiation of ECMO treatment to conversion was 6.9 (3.7–14) days (ranging from 21.5 h to 41 days).

The main indications for conversion were circulatory shock (*n* = 12) and RVF (*n* = 30, of which 15 also had circulatory shock). The remaining four patients were converted to help decrease intracranial pressure (*n* = 1), pulmonary edema caused by a mitral valve prolapse (*n* = 1), and unknown (*n* = 2). Subsequently, to avoid selection bias, these four patients were not included in the detailed analyses of RVF and circulatory failure below. The mortality rate for the converted group was significantly higher compared to non-converted patients (61% vs. 16%, *p* < 0.001), with conversion inferring a relative risk (RR) for mortality of 3.76 (95% CI 2.48–5.71, *p* < 0.0001). Detailed data for all 46 patients at the time of conversion and their outcomes are presented in Supplemental Digital File Table S2.

### 3.1. Subgroup Analyses: Converted Patients

For the twelve patients who were converted due to circulatory shock (without RVF), the time to conversion was 2.1 (1.3–4.1) days. The corresponding time for the RVF-group was 11 (5.4–15) days (significantly longer (*p* < 0.001)), indicating two different courses of development. Of note, both groups were converted within one day of recognition of the need for circulatory support (0 (0–0.5) days in circulatory failure vs. 0.9 (0–1) days in RVF (*p* = 0.12)). None of the patients who developed circulatory failure were diagnosed with a myocardial infarction (MI).

The organ failure score, SOFA, increased from 12 (9–13) at admission to 15 (13–17) at conversion (*p* < 0.001). SOFAc and number of vasoactive drugs also increased, from 43 (3–4) to 4 (3–4) (*p* < 0.05), and 1 (1–1) to 21 (1–2) (*p* < 0.00021), respectively. VIS, which decreased during the first 12 h (*p* < 0.02), pivoted and increased significantly until the time of conversion (Table 2). The most commonly used vasoactive drugs before ECMO were norepinephrine (79%, 33/42) and epinephrine (10%). At conversion, norepinephrine was used in 90%, milrinone in 40%, and epinephrine in 21% of the patients. The RESP score on admission for the converted cohort (−3 (−4.8–1)) corresponded to an estimated mortality rate (EMR) of ~65% (95% CI 53%–75%), while the SAVE score at the time for conversion for these patients (−8 (−10–−5.3)) corresponded to an EMR of ~74% (95% CI 67%–82%). These estimations of EMR and their clinical trajectory indicate that the patients had deteriorated from admission to the time of conversion (i.e., their estimated mortality had increased). The converted patients who died also had significantly lower admission RESP scores compared to those who survived (−4 (−5.8–−0.3) vs. 0 (−2.3–3), *p* = 0.012), (Table 3).

### 3.2. Right Ventricular Failure

Mortality for patients who were converted due to RVF without circulatory shock was 67% (10/15), which was the same as in patients with RVF and circulatory shock (67%, 10/15). Circulatory shock patients (in the absence of RVF) had a mortality rate of 50% (6/12). No increase in RR for death was seen in patients converted due to RVF as compared to those patients in circulatory shock without RVF, (RR 1.33 (95% CI 0.72–2.48), *p* = 0.46). However, converted patients with RVF showed a significantly longer time on ECMO compared to those in circulatory failure, 29.6 (17.3–42.1) vs. 10.4 (5.9–29.1) days (*p* = 0.048), respectively. Paradoxically, on admission, patients who later developed RVF presented with a significantly lower VIS, lactate, and SOFA_in_ score than those who developed circulatory failure (Table 4). Besides the more rapid development of the illness in the circulatory shock patients, these required higher doses of circulatory support and experienced higher lactate at conversion. The RVF patients showed a trajectory of increased SOFA score from admission to RVF diagnosis (11 (8.3–12) to 14 (12.3–16.8), *p* < 0.001)). Furthermore, their admission RESP score was higher than their pre-conversion SAVE score (−3 (−4–0.8) vs. −8 (−10–−6)). This indicates that they had deteriorated, as the corresponding EMR was 64% (95% CI 56–72%) for the RESP score and 74% (95% CI 67–82%) for the SAVE score. However, it should be noted that RESP and SAVE scores are not interchangeable when it comes to estimated risk to survival.

## 4. Discussion

The aim of this study was to present our institutional experience from a population-based cohort of adult patients who required conversion from VV to VA ECMO. Out of 213 patients, 21% required conversion to VA ECMO, a frequency higher than previously reported [2,15]. This may be owing to the fact that our unit is a dedicated ECMO center that had offered all modes of support (VV, VA, and VVA) to adults for almost three decades. Our close monitoring of RV function comes from lessons learned 20 years ago, when “total lung rest” was practiced or the ventilatory pressures were markedly reduced with risk of lung collapse, running the risk of hypoxic vasoconstriction and increased right ventricle afterload [9,10,20]. However, our current practice is based on a concept favoring maintained tidal volumes [20] (Table 1), early tracheostomy, and awake patients with spontaneous triggering with reduced driving pressure and maintained PEEP. Nonetheless, signs indicating RV impairment at admission or later during the course of ECMO support are important to notice and to follow.

Compared to non-converted patients, converted patients had increased in-hospital mortality. This was also implied by the lower admission RESP score (indicating higher estimated mortality). Somewhat worryingly, the mortality rate for the patients who were converted from VV to VA ECMO was also higher than the overall mortality rate for VA ECMO patients at our center (61% vs. 36%) [16]. This suggests that there were cases where delayed recognition of circulatory failure may have increased mortality in VV ECMO patients. This stresses the value of regular circulatory evaluation in VV ECMO patients in order to identify those who may have an impending need for circulatory support and where earlier conversion to VA ECMO might improve survival. Some patients may indeed benefit from being commenced on VA ECMO from the start.

Patients converted due to circulatory shock were identified as candidates for conversion within 2 days of ECMO initiation, whereas those who developed RVF were recognized after 11 days. RVF was also the most common indication for conversion to VA ECMO. The RVF subgroup who did not develop circulatory shock were in this early phase of circulatory failure undistinguishable from the non-converted VV patients (RESP 0; SAPS-3 74.2 vs. RESP 1; SAPS-3 74.1, respectively). This further stresses the importance of continued daily monitoring of organ and cardiac function to help identify the patients who will develop RVF during treatment. However, the RVF subgroup who developed circulatory failure significantly differed in admission RESP score compared to the whole VV cohort, suggesting that these patients were in a more severe state of illness already on admission. The confidence intervals of RESP “−3” and “1” *did not* overlap, indicating a probable absolute mortality risk difference of approximately 20%. This increased risk of death in patients with RVF has also been described in conventional intensive care patients not supported with ECMO [31].

For all converted patients, SOFA scores increased from admission to the time of conversion by ~3 points. This corresponds to a >20% increased risk of mortality in that section of the SOFA score scale [33]. A similar observation of increased EMR was observed when comparing SAVE scores at conversion to admission RESP scores (although the SAVE and RESP scores are not interchangeable, both scores consider “0 points” as an EMR of 50%, and a score >0 indicates a higher chance for survival and vice versa). Thus, we found that converted patients had a temporal trajectory of both increased SOFA score and EMR obtained from RESP and SAVE scores between admission and conversion. This suggests that temporal trajectories of these scores, as well as daily echocardiography, could be used for early identification of patients who require conversion. Thereby avoiding an emergent conversion with fully developed circulatory shock or cardiac arrest.

## 5. Study Limitations

Limitations of this study include the retrospective design and the use of high-volume center data for generalized recommendations to ECMO providers. No patients with circulatory failure suffered a MI. However, cytokines were not assessed, and we can therefore only speculate on cytokine/inflammatory influence on myocytes and vascular walls. While the study spanned over a 14-year period, the treatment and management protocols have been rather uniform during this time. We consider the exclusion of patients converted within the first 6 h after commencement of ECMO a reasonable way to avoid study bias since such an early conversion was presumably due to poor selection of the initial mode.

## 6. Clinical Implications

The benefit of blood oxygenation achieved by VV ECMO is meaningless if the oxygen is not delivered to the tissue. Consequently, patients with respiratory illness and signs of shock or circulatory failure run the risk of deterioration even if VV ECMO is applied. If a patient on VV ECMO develops a need for circulatory support, the benefits of conversion to VA ECMO must be weighed against the risks involved in cannula re-configuration. As highlighted in this study, patients converted from VV to VA ECMO had a higher mortality rate than those not converted. Notably, this mortality was also higher than for patients who commenced and were treated with VA ECMO. These results, together with the results from a recent publication [16], suggest that there was a proportion of patients who are commenced on VV ECMO and in whom a delayed recognition of the need for circulatory support increased their risk of death. These patients might have been better off if they were treated with VA ECMO from the start. In other words, the concept of “respiratory ECMO” should not be synonymous with VV ECMO per se, but rather the modality required to support organ tissue oxygenation in an illness categorized as respiratory. This modality could be either VV, VA, VVA, or veno-pulmonary.

The subsequent question is which patients should be started on VA instead of VV ECMO? Our results showed that patients who required conversion were identified using routine echocardiography but could also be predicted by their lower on-admission RESP scores and temporal deterioration in daily SOFA scores. Thus, it is imperative that VV ECMO patients who do not show significant recovery within the first 6–12 h, are considered at-risk for later VA ECMO requirements and undergo echocardiography exams and daily risk score assessments (e.g., SOFA) to identify early signs of circulatory dysfunction. Naturally, in accordance with recommendations made by others [8,15], we agree that VV ECMO is the suitable modality in patients without shock. However, in patients who show signs of shock, both with or without RVF, VA ECMO should be the modality of choice. If ECMO is considered in a patient where no cardiac output monitoring device or echocardiographic competence is available prior to cannulation, the safest choice of modality may be VA ECMO with a jugulo-femoral cannulation configuration [32,34,35]. We have used the jugulo-femoral VA configuration for almost three decades. VVA has been used in approximately 3% of our adult cases over the years. Our preference for VA has proven to be a winning concept [16]. It is simple and provides adequate oxygen delivery to the brain and coronaries in almost all patients, including those with severe lung failure [16,30]. Concerning the more complex VVA circuits, Y-piece connectors and gate-clamps (to balance ‘V’ and ‘A’ support) carry an increased risk of platelet activation and hemolysis [36]; the return to two different pressurized compartments the respective cannula’s differential pressure influences flow momentarily which also limits perfusion of the cannulated leg; and the introduction of recirculation by the VV component [34]. In the rare case of VVA, we apply a 15 Fr/50 cm lighthouse tip cannula via either femoral vein for return to reduce flow by resistance (to avoid the gate-clamp).

Lastly, while more studies are needed to elucidate the value of ECMO patients being converted from VV to VA ECMO, we believe that ECMO providers who only offer VV ECMO support should consider developing routines for or collaboration with a center that has experience with VA ECMO.

To summarize:

- At least 20% of patients subjected to respiratory support with ECMO may need combined cardio-respiratory support, i.e., VA ECMO from the start or during the course of treatment.
- Early realization of the need for VA ECMO probably improves patient prognosis, in the best case decision for VA is made already on admission. These patients may be identified on admission by a negative (-) RESP score. These patients need daily assessments.
- Patients converted to VA due to cardio-circulatory failure during VV ECMO are usually recognized within the first days.
- Patients who develop RVF are more difficult to recognize since typical features take up to two weeks to be present clinically. These patients cannot be distinguished from the general VV population at the time of admission for ECMO. Daily organ function assessments, including echocardiography and the trend of vasoactive support, are important tools to identify these subjects earlier.

## 7. Conclusions

We found that patients converted from VV to VA ECMO had a higher mortality rate compared to non-converted patients and compared to our historical VA ECMO cohort. We argue that VV ECMO should not be the default modality in “respiratory failure” in cases with signs of circulatory impairment. In these subjects, our findings suggest that evaluation, including a thorough assessment of the RV, is necessary, and in uncertainties of RV function VA ECMO should be considered. Admission RESP scores, daily echocardiography, and SOFA trajectories can be used for early recognition of VV patients who might need conversion to VA ECMO. Multi-centric studies are warranted to validate these findings.

## Figures and Tables

**Table 1:** Converted and non-converted patients. Baseline and outcome data for non-converted and converted venovenous extracorporeal membrane oxygenation patients. This table includes all 46 patients. * RESP score at the time of decision for ECMO; ** Tidal volume for the non-converted group was based on the lowest volume noted during ECMO treatment, while the tidal volume for the converted group was based on the lowest volume at the time of diagnosis and when the decision for conversion was made. Data was collected from digital medical charts.; # missing or incomplete medical charts, *n* = 6. Abbreviations: Circ.: circulatory; ECMO: extracorporeal membrane oxygenation; RESP: Respiratory Extracorporeal membrane oxygenation Survival Prediction score; RVD: right ventricular dysfunction; RVF: right ventricular failure; SAPS-3: Simplified Acute Physiology Score.

	Non-Converted	Converted	*p*-Value
Number, n	167 (missing *n* = 6)	46	
Male sex, %	66	65	1.0
Age, years	49 (34.5–59)	50 (33–60)	0.74
SAPS-3	74 (67–81)	72 (67–80)	0.87
RESP *	2 (0–4)	−2.5 (−4–1)	<0.0001
Tidal volume **	288 (150–400)	200 (55–388) #	0.11
RVD, %	28	87	<0.0001
RVF, %	10	72	<0.0001
Circ. failure, %	51	86	<0.0001
Circ. Shock, %	49	44	0.62
Days on ECMO, *n*	6.6 (3–12)	22 (12–40)	<0.0001
Mortality rate, %	16	61	<0.0001

**Table 2:** Patients converted from venovenous to venoarterial extracorporeal membrane oxygenation. Respiratory and circulatory data at admisson, after 12 h of venovenous extarcorporeal membrane oxygenation (ECMO) support, and at the time of conversion to venoarterial support. # Six patients (13%) had arterial blood gases available 12 h after ECMO initiation, and 14 patients (30%) had arterial blood gases available in the last 4 h before conversion to VA ECMO. Clinical monitoring was made from pre-oxygenator blood gas assessments not presented in this table. Abbreviations: FiO2: fraction inspired oxygen; PIP: peak inspiratory pressure; PEEP: positive end-expiratory pressure; Vt: tidal volume, BW: body weight; RR: respiratory rate; BP: blood pressure; Hbg: hemoglobin concentration; SOFAc: sequential organ failure assessment—score for the circulatory domain; VIS: vasoactive inotropic score (reference [24]).

	At Admission (Pre-ECMO)	VV ECMO 12 h	*p*-Value (Pre-ECMO vs. 12 h)	VV Data before Conversion to VA	*p*-Value (Pre-ECMO vs. Conv.)	*p*-Value (12 h vs. Conv.)
pH	7.28 (7.18–7.35)	7.38 (7.34–7.45)	<0.0001	7.38 (7.31–7.41)	<0.0001	
pCO_2_, kPa	7.7 (6.2–8.6)	5.5 (5.5–5.65)	#	5.3 (4.8–5.8)	#	
PpreCO_2_, kPa	--	5.8 (5.3–6.4)	--	5.6 (5.1–8)	--	
pO_2_, kPa	7.0 (6.2–8.5)	7.4 (6.0–7.8)	#	6.1 (4.9–8.0)	#	
PpreO_2_, kPa	--	5.9 (5.5–6.4)	--	5.4 (4.8–5.8)	--	<0.01
SpO_2_, %	84 (74–88)	88 (79–93)	<0.02	80 (71–88)		<0.001
Lactate, mmol/L	2.3 (1.7–4.3)	2.1 (1.4–3.4)		1.8 (1.3–3.4)		
FiO_2_, %	100 (100–100)	50 (40–60)	<0.0001	60 (50–70)	<0.0001	<0.001
PIP, cmH_2_O	35 ± 7.7	27 ± 4.9	<0.0001	24 ± 4.6	<0.0001	
PEEP, cmH_2_O	14 ± 4.3	8.8 ± 3.2	<0.0001	6.4 ± 3.6	<0.0001	<0.01
Driving pressure, cmH_2_O	20 (16–24)	18 (14–21)		18 (15–21)		
Vt, mL	473 ± 159	315 ± 210	<0.0002	188 ± 155	<0.0001	<0.003
Vt/kg BW, mL/kg	6.1 (5.2–7.3)	3.5 (1.8–5.6)	<0.0001	1.8 (0.8–3.8)	<0.0001	<0.0001
RR, min^−1^	25 ± 7.6	20 ± 6.0	<0.02	24 ± 10		
Mean arterial BP, mmHg	70 (62–80)	68 (63–73)		66 (61–72)		
Hgb, g/L	111 ± 15.5	115 ± 11.4		118 ± 13.0	<0.02	
ECMO blood flow, L/min	--	4.3 (3.85–4.8)		4.5 (4.1–5.0)		
DecmoO_2_, mL/min	--	224 ± 46	--	243 ± 42	--	<0.02
SOFAc	3 (3–4)	3 (0–4)	<0.02	4 (3–4)	<0.05	<0.002
No. vasoactive drugs, n	1 (1–1)	1 (0–1)	<0.05	2 (1–2)	<0.001	<0.0001
VIS	12 (5–42)	5 (0–20)	<0.02	19 (10–29)		<0.001

**Table 3:** Comparison between survivors and deceased. Variables associated with mortality in patients converted from venovenous to venoarterial extracorporeal membrane oxygenation. Data presented as mean ± SD or median (IQR). Differences for variable trajectories within same group: * *p* = 0.020; ** *p* = 0.013; # *p* = 0.007; † *p* < 0.001. Abbreviations: ECMO: extracorporeal membrane oxygenation; FiO2: fraction inspired oxygen; MAP: mean arterial blood pressure; PEEP: positive end-expiratory pressure, PIP: peak inspiratory pressure; RESP: Respiratory Extracorporeal membrane oxygenation Survival Prediction score; RVF: right ventricular failure; SAPS-3: Simplified Acute Physiology Score; SAVE: Survival After Venoarterial ECMO score; SOFA: Sequential Organ Failure Assessment score; SOFAconv: SOFA score before conversion; SOFAdelta: SOFAconv–SOFAin; SOFAin: SOFA score before ECMO; SOFAc: SOFA score for the circulatory domain; VIS: vasoactive inotropic score (reference [24]); Vt: tidal volume.

	Alive (*n* = 16)	Deceased (*n* = 26)	*p*-Value
pre-ECMO data			
Male sex, %	69	65	1.0
Age, years, (range)	47.5 ± 17.5	48.6 ± 16.7	0.39
	(19–67)	(23–77)	
Body weight, kg	76 (72–84)	77 (70–86)	0.77
pH	7.30 (7.21–7.34)	7.26 (7.18–7.34)	0.92
pCO_2_, kPa	7.7 (5.8–8.6)	8.0 (6.7–8.6)	0.47
pO_2_, kPa	7 (6.4–9)	7 (6.3–7.6)	0.89
SaO2, %	83 (71–89)	84 (75–87)	0.96
FiO_2_, %	100 (97.5–100)	100 (100–100)	0.045
PIP, cmH_2_O	35 ± 8.3	35 ± 7.8	0.76
PEEP, cmH_2_O	14 ± 4.3	14 ± 4.4	0.86
Vt, mL	481 ± 199	463 ± 133	0.74
MAP, mmHg	78 (64–81)	70 (63–80)	0.65
Hemoglobin, g/L	113 ± 14	109 ± 17	0.44
Lactate, mmol/L	2 (1.1–4.6)	2.4 (1.8–3.4)	0.20
No. of vasoactive drugs, n	1 (1–1) *	1 (1–1) **	0.53
VIS	10 (9–40)	10.5 (4–44)	0.94
SOFAin	11.5 (9–12.3) #	12 (8.3–13.8) †	0.88
SOFAc	3 (2–4)	3.5 (3–4)	0.35
SAPS-3	71.4 ± 10.0	76.4 ± 12.9	0.20
RESP score	0 (−2.3–1)	−4 (−5.8–−0.3)	0.012
**Data at time of conversion to VA ECMO**			
Time to conversion, days, (range)	8.4 (2.1–15)	6.9 (3.9–13.8)	0.68
	(0.9–31.3)	(1.1–33.8)	
Time from recognition of RVF or circulatory shock to conversion, days, (range)	0.6 (0.0–1.0)	0 (0–0.8)	0.92
	(0–8)	(0–3)	
pH	7.37 (7.30–7.40)	7.38 (7.32–7.42)	0.41
SaO_2_; SpO_2_, %	82 (70–86)	77 (71–88)	0.95
FiO_2_ (%)	60 (50–70)	60 (50–60)	0.78
PIP, cmH_2_O	24 ± 5.6	25 ± 4.2	0.49
PEEP, cmH_2_O	6.2 ± 4.2	6.1 ± 3.4	0.92
Vt, mL	183 ± 143	180 ± 156	0.95
MAP, mmHg	66 (64–71)	67 (60–73)	0.75
Hemoglobin, g/L	117 ± 16	118 ± 11	0.86
ECMO blood flow, L/min	4.8 (4–5.3)	4.4 (4.1–5)	0.71
Lactate, mmol/L	1.5 (1.3–2.1)	1.7 (1.2–3.8)	0.07
No. of vasoactive drugs, *n*	2 (1–2.2)*	1 (1–2) **	0.45
VIS	26 (11–31)	14 (6–26)	0.16
SOFAconv	14 (12.8–16.3)#	15 (14–17) †	0.23
SOFAdelta	2.5 (1–4)	3 (2–6)	0.25
SOFAc	4 (3–4)	4 (3–4)	0.70
SAVE score at conversion	−6.5 (−10–−4)	−9 (−10.8–−7)	0.08
Days on ECMO, *n*	23.3 (13.1–45.9)	25.5 (12.1–40.1)	0.78

**Table 4:** Circulatory shock and right ventricular failure. Data for the groups converted from venovenous to venoarterial extracorporeal membrane oxygenation due to circulatory shock or right ventricular failure (RVF). The right part of the table presents the subgroups of RVF with concomitant shock or RVF with no circulatory shock. Normally distributed data are presented as mean ± SD, and non-parametric data as median (IQR). Differences for variable trajectories within same group: * *p* = 0.052; ** *p* < 0.01; # *p* = 0.014; † *p* < 0.0001; ‡ *p* = 0.028; $ *p* < 0.0001. Abbreviations: ECMO: extracorporeal membrane oxygenation; FiO2: fraction inspired oxygen; MAP: mean arterial blood pressure; PEEP: positive end-expiratory pressure; PIP: peak inspiratory pressure; RESP: Respiratory Extracorporeal membrane oxygenation Survival Prediction score; RVF: right ventricular failure; SAPS-3: Simplified Acute Physiology Score; SAVE: Survival After Venoarterial ECMO score; SOFA: Sequential Organ Failure Assessment score; SOFAconv: SOFA score before conversion; SOFAdelta, (=SOFAconv-SOFAin); SOFAin: SOFA score before ECMO; SOFAc: SOFA score for the circulatory domain; VIS: vasoactive inotropic score (reference [24]); Vt, tidal volume; *w*, with; *w/o*, without.

	Circulatory Shock (*n* = 12)	*p*-Value (Circ Shock vs. RVF all)	RVF all (*n* = 30)	RVF Presented with Shock (*n* = 15)	RVF not Presented with Shock (*n* = 15)	*p*-Value (RVF, *w* vs. *w*/*o* Shock)
pre-ECMO data in patients who later were converted to VA ECMO						
Male sex, %	67	1.0	67	53	80	0.25
Age, years, (range)	44.9 ± 19.5	0.66	47.5 ± 16.2	47.5 ± 13.7	47.6 ± 18.8	0.98
	(19–74)		(19–78)	(19–66)	(22–78)	
Body weight, kg	74 (69–82)	0.44	77 (71–86)	73 (66–82)	83 (77–89)	0.08
pH	7.21 (7.18–7.32)	0.20	7.31 (7.21–7.37)	7.34 (7.24–7.36)	7.29 (7.19–7.36)	0.52
pCO_2_, kPa	7.9 (6.4–8.1)	0.60	7.9 (6.2–8.8)	8.1 (6.4–8.7)	7.6 (6.1–9)	0.60
pO_2_, kPa	7.1 (6.3–7.8)	0.71	7.0 (6.2–8.5)	6.8 (6–8.1)	7.2 (6.7–8.5)	0.34
SaO_2_, %	80 (70–84)	0.14	84 (75–88)	84 (76–89)	84 (78–87)	0.90
FiO_2_, %	100 (100–100)	0.77	100 (100–100)	100 (100–100)	100 (100–100)	0.92
PIP, cmH_2_O	35 ± 4.6	0.76	35 ± 9.1	35 ± 11	35 ± 7.7	0.93
PEEP, cmH_2_O	15 ± 4	0.32	14 ± 4.4	12 ± 4.7	14 ± 4.0	0.22
Vt, mL	491 ± 194	0.60	461 ± 145	495 ± 125	431 ± 158	0.26
MAP, mmHg	61 (58–74)	0.006	78 (69–86)	71 (65–90)	80 (73–82)	0.64
Hemoglobin, g/L	120 ± 14	0.01	107 ± 15	112 ± 16	101 ± 12	0.06
Lactate, mmol/L	4.4 (2.4–6.6)	0.002	2.1 (1.2–2.4)	2.3 (1.2–3.0)	1.9 (1.2–2.2)	0.37
No. of vasoactive drugs, *n*	1(1–1) *	0.22	1(1–1) **	1 (1–1)	1(1–1)	0.96
VIS	36 (9–60)	0.04	9.7 (3–33)	11 (3.4–38)	9.4 (2.6–12)	0.54
SOFAin	13 (12–14.5) #	< 0.0001	11 (8.3–12) †	11 (9–12.5) ‡	11 (8–12) $	0.53
SOFAc	4 (3–4)	0.15	3 (0.5–4)	3 (0–4)	3 (2.5–4)	0.59
SAPS-3	76.3 ± 15.0	0.53	73.7 ± 10.8	75.3 ± 10.8	72.1 ± 11.0	0.42
RESP score	−2 (−6–3.3)	0.61	−3 (−4–0.8)	−3 (−5.5–−1.5)	0 (−4–1)	0.026
**Data at time of conversion to VA ECMO**						
Time to conversion, days, (range)	2.1 (1.3–4.1)	<0.001	11.1 (5.4–15)	12.3 (4.1–15)	10.4 (6.6–17.2)	0.98
	(1–18.9)		(1–40.9)	(1–40.9)	(3.8–34.2)	
Time from recognition of RVF or circulatory shock to conversion, days, (range)	0 (0–0.5)	0.12	0.9 (0–1)	1 (0.4–1.5)	0.7 (0–1)	0.058
	(0–2)		(0–8)	(0–8)	(0–1.6)	
pH	7.33 (7.29–7.39)	0.12	7.39 (7.33–7.42)	7.40 (7.34–7.44)	7.37 (7.34–7.40)	0.21
SaO_2_; SpO_2_, %	85 (76–90)	0.11	77 (70–85)	78 (74–88)	73 (66–82)	0.18
FiO_2_, %	60 (60–72)	0.17	50 (50–62)	50 (50–60)	60 (50–65)	0.30
PIP, cmH_2_O	25 ± 4.9	0.47	24 ± 4.7	24 ± 2.9	25 ± 5.8	0.57
PEEP, cmH_2_O	8.6 ± 4.3	0.005	5.1 ± 2.8	4.9 ± 2.9	5.2 ± 2.8	0.80
Vt, mL	216 ± 142	0.37	166 ± 152	178 ± 132	156 ± 172	0.61
MAP, mmHg	67 (64–72)	0.87	66 (60–73)	66 (56–71)	70 (64–74)	0.16
b-Hemoglobin, g/L	114 ± 17	0.18	119 ± 11	122 ± 9	116 ± 12	0.02
ECMO blood flow, L/min	4.5 (4–5.1)	0.73	4.5 (4.2–5.1)	4.5 (4–4.8)	5.1 (4.2–5.5)	0.07
p-Lactate, mmol/L	3.8 (2.2–7.0)	< 0.0001	1.4 (1.1–1.8)	1.2 (1.1–1.4)	1.7 (1.4–2.5)	0.02
No. of vasoactive drugs, *n*	2 (1–3) *	0.21	1 (1–2) **	1 (1–2)	1(1–2)	0.07
VIS	29 (23–52)	0.006	12 (6–26)	10 (4.5–13)	18 (10.5–27)	0.06
SOFAconv	16 (14.8–18) #	0.07	14 (12.3–16.8) †	13 (11.5–14.5) ‡	15 (14–17) $	0.07
SOFAdelta	2.5 (1–4.3)	0.61	3 (1–5.8)	3 (0.5–4)	4 (2–7.5)	0.09
SOFAc	4 (4–4)	0.29	4 (3–4)	3 (3–4)	4 (3–4)	0.07
SAVE score at conversion	−9.5 (−10.5–−4.8)	0.86	−8 (−10–−6)	−7 (−10–−6)	−8 (−12–−6)	0.47
Days on ECMO, *n*	10.4 (5.9–29.1)	0.048	29.6 (17.3–42.1)	34.3 (22.2–46.9)	22.8 (13–33.9)	0.09
Mortality rate, %	50	0.48	67	67	67	1.0

## Data Availability

The datasets generated and/or analyzed during the current study are not publicly available due to patient secrecy but are available from the corresponding author on reasonable request.

## Supplementary Materials

The following are available online at https://www.mdpi.com/2077-0375/11/3/188/s1. Table S1: Demographic and admission data. Table S2: Data and outcome after conversion from venovenous to venoarterial extracorporeal membrane oxygenation.
