# Supplementary material for: Conversion from Venovenous to Venoarterial Extracorporeal Membrane Oxygenation in Adults
Source: Membranes (Basel). 2021 Mar 9;11(3):188. doi: 10.3390/membranes11030188 (PMC7999389; doi:10.3390/membranes11030188)
Supplement: Supplementary file 1 [file membranes-11-00188-s001.pdf]

Supplemental additional data Table S1

Conversion from Venovenous to Venoarterial Extracorporeal Membrane Oxygenation in Adults

Lars Falk[1,2], Alexander Fletcher-Sandersjöö[3,4], Jan Hultman[1,2], Lars Mikael Broman[1,2]

1) ECMO Centre Karolinska, Department of Pediatric Perioperative Medicine and intensive Care, Karolinska University Hospital, Stockholm, Sweden; 2) Department of Physiology and Pharmacology, Karolinska Institutet, Stockholm, Sweden; 3) Department of Clinical Neuroscience, Karolinska Institutet, Stockholm, Sweden; 4) Department of Neurosurgery, Karolinska University Hospital, Stockholm, Sweden

Table S1: Demographic and admission data

Patient group submitted to venovenous extracorporeal membrane oxygenation (ECMO) that were converted to venoarterial ECMO after at least six hours from start of extracorporeal life support. The four patients marked in italic were not included in the detailed analyses of RVF and LVF. The indications for conversion in these subjects were unknown (n=2), increased intracranial pressure (n=1), and pulmonary oedema due to a mitral valve prolapse (n=1).

Pat no.	Age years	Sex	BW kg	Diagnosis	Trnsp on ECMO	pH	pCO2 kPa	pO2 kPa	SaO2 %	Hgb g/L	Lact mmol/L	PIP cmH2O	PEEP cmH2O	Vt mL	MAP mmHg	SAPS 3	RESP	SOFA in	SOFA circ	No. vasoactive drugs	VIS	Vasoactive drug/s used
**1**	19	f	55	ARDS trauma/ postop	yes	7.34	8,3	9.5	94	104	0.6	HFOV	HFOV	HFOV	100	63	-2	9	3	1	90	Nor
**2**	47	m	85	Oesophagus ca	yes	7.27	12	6.2	70	101	1.1	HFOV	HFOV	HFOV	71	72	-5	12	4	1	10	Nor
**3**	57	m	96	Pneumoccal pneu	yes	7.37	8.5	6.8	72	146	2.1	42	18	750		66	0	11	4	1	31	Nor
**4**	57	f	88	Wegener granulomatosis	no	7.47	4.9	7.1	87	107	3	51	10	447	111	61	-7	7	0	0	0	
**5**	30	f	68	Pneumonia unspec	yes	7.43	6	7.4	90	81	1.7	HFOV	HFOV	HFOV	90	68	-4	9	3	1	4,4	Nor
**6**	53	m	65	Stone lung, ca	no	7.36	6.6	6.7	84	106	2.3	28	5	570	65	67	-6	13	0	1	12,3	Dopa
**7**	62	m	74	Trauma bilat fem fract	no	7.18	8.2	3.2	35	144	6.2	37	14	636	68	58	-7	13	3	1	6,3	Nor
**8**	33	f	73	Aspiration	no	7.34	8.7	6.7	88	120	1.2	44	16	288	74	69	-3	8	0	1		Dobu
**9**	44	f	58	Amyloidos, resp fail	yes	7.32	7.5	10.3	88	98	1.3	30	11	341	63	78	-10	10	3	1	9,2	Nor
**10**	46	f	72	Hypoxemia psot liverTX	yes	7.40	6.1	5.5	71	103	0.9	16	6	600	62	68	1	10	0	0	0	
**11**	47	m	65	ARDS non-trauma	yes	7.23	15.8	7.0	76	120	2.4	55	12	410	95	92	-3	8	0	0	0	Nor
**12**	66	m	78	ARDS non-trauma	yes	7.26	6.9	9.8	96	114	2.3	34	16	460	90	79	-5	11	3	1	3	Nor
**13**	60	m	95	Pneumonia bact	yes	7.11	8.1	4.4	83	110	19	30	7	580		88	-3	12	3	1	13	Nor
**14**	57	m	80	Pneumonia bact	yes	7.35	5.9	11.4	96	113	2.0	45	20	620	80	82	-3	16	4	1	38	Nor
**15**	51	m	82	Pneumonia virus	yes	7.32	8	7.3	70	104	2.4	33	20	540	60	60	4	8	3	1	4	Nor
**16**	27	f	77	Pneumonia virus	yes	7.31	4.6	6.9	65	101	5.6	37	20	400	77	62	6	9	3	1	9,4	Nor
**17**	57	m	79	Aspiration	yes	7.20	8.8	7.7	88	115	1.1	33	15	480	80	78	1	6	0	0	0	
**18**	22	m	77	Pneumonia virus	yes	7.23	9.4	7.2	80	120	1.0	48	20	510	87	68	3	11	3	1		Nor
**19**	49	m	93	Pneumonia virus	yes	7.29	5.2	8.4	86	110	2.2	29	17	700	102	60	3	8	0	0	0	
**20**	60	m	83	Pneumonia bact	yes	7.23	7.8	5.2	71	118	2.3	31	8	499	78	82	-1	14	3	1	5,6	Nor

**21**	51	m	65	Aspiration	yes	7.15	8.9	7.6	91	95	8.8	35	20	252	65	74	-3	14	4	1		Nor
**22**	68	f	50	Pneumonia bact	yes	7.26	7.2	5.3	66	128	3.7	25	14	550	61	74	1	13	4	2	25	Nor, (+Epi boluses)
**23**	33	m	86	Pneumonia bact	yes	6.97	23.1	11.8	93	115	2.6	51	20	370	81	70	-4	12	4	2	46	Nor, Epi
**24**	22	m	60	Aspiration	no	6.76	12.5	6.8	69	145	6.3	37	14	330	55	65	-6	13	0	1	15,6	Nor
**25**	39	f	90	Pneumonia bact	yes	7.32	8.1	15.1	98	98	1.8	HFOV	HFOV	HFOV	83	70	0	8	2	1	1,85	Dobu
**26**	63	m	120	Pneumonia bact	yes	7.15	8	6.7	78	120	7.3	37	15	864	55	67	0	12	4	1	35	Nor
**27**	24	m	120	ARDS trauma/ postop	yes	7.42	12.1	7.0	84	98	1.0	38	18	290	70	50	-8	9	3	1	5	Nor
**28**	19	m	86	Pneumonia bact	yes	7.30	6.6	4.3	61,6	118	4.3	36	12	663	80	72	6	12	4	2	51	Nor, Mil
**29**	53	m	75	Pneumonia bact	no	7.18	7.3	7.4	86	111	2.4	34	16	390	80	97	1	16	4	1	38	Nor
**30**	77	m	86	Pneumonia bact	yes	7.39	6.7	6.8	80	105	2.0	28	8	524	60	78	-3	5	0	0	0	
**31**	74	f	63	Sepsis	no	7.18	7.4	6.9	83	117	4.3	31	14	230	60	100	-7	13	4	1	60	Nor
**32**	56	m	74	Pneumonia bact	yes	7.40	5.7	7.3	86	126	1.6	28	15		70	60	5	8	3	1	10	Nor
**33**	32	f	71	Pneumonia virus	yes	7.35	5.2	5.8	77	105	3.1	28	19	470	70	80	-1	14	4	2	53	Nor, (+Epi boluses)
**34**	64	m	70	Sepsis	yes	7.23	7.6	8.5	74	96		36	12	427	80	80	-4	14	3	1	10	Nor
**35**	67	m	71	Pneumonia bact	yes	7.47	4.3	4.9	74	99	2.1	28	12	90	85	68	1	12	3	1	43	Nor
**36**	32	m	95	ARDS non-trauma	yes	7.44	5.4	6.2	84	79	2.2	36	11	690	76	68	-4	11	4	1	11	Nor
**37**	36	f	50	ARDS non-trauma	yes	7.16	9.3	6.6	82	99	1.4	35	12	300	65	78	-4	13	4	1	12	Nor
**38**	59	m	76	Pneumonia bact	yes	7.13	8.6	5.4	75	141	3.4	33	12	564	60	83	-8	14	4	2	70	Nor, Epi
**39**	58	f	70	ARDS non-trauma	yes	7.19	8.3	9.1	88	127	12.7	29	11	280		91	-6	14	4	1	60	Nor
**40**	78	m	81	Pneumonia bact	yes	7.31	6.9	5.4	75	86	1.8	23	12	500	70	72	1	7	4	1	6,6	Nor
**41**	68	m	75	Sepsis	yes	7.08	11	8.8	91	123	5.6	32	16	470	65	96	-1	16	4	1	40	Nor
**42**	47	f	90	Pneumonia bact	yes	7.30	6.9	8.5	86	108	2.0	36	20	564	67	66	4	9	4	2	35	Nor
**43**	48	f	70	Pneumococcla sepsis	no	7.18	8.1	9.5	84	99	10.2	35	15	395	61	99	3	16	4	1	70	Nor
**44**	56	m	85	ARDS non-trauma	yes	7.16	8.6	11.1	93	89	0.9	42	10	360	80	82	0	12	4	3	9,3	Nor, (+Epi ?), Mil
**45**	67	m	74	Pulmonary edema	yes	7.20	5.3	6.0	75	119	2.0	34	14	674	56	74	-2	12	4	1	60	Nor
**46**	39	f	65	Pneumonia bact	yes	7.33	5.2	7.3	84	111	4.5	36	22	346	55	80	-3	16	4	2	92	Nor, Mil

Abbreviations: ARDS, acute respiratory distress syndrome; bact., bacterial; ca, cancer; fract, skeletal fracture; resp fail, respiratory failure; TX, transplant; f, female; m, male; BW, body weight; Lact, plasma lactate; FiO2, fraction inspired oxygen; PIP, peak inspiratory pessure; PEEP, positive end-exppiratory pressure; Vt, tidal volume; HFOV, high frequency oscillation ventilation; MAP, mean arterial blood pressure; Hgb, hemoglobin concentration; SAPS-3, Simplified Acute Physiology Score revision 3; SOFA in, Sequential Organ Failure Assessment score at admission; SOFA circ, SOFA score for circulatory domain; VIS, vasoactive inotropic score; Nor, norepinephrine; Dopa, dopamine; Dobu, dobutamine; Epi, epinephrine; Mil, milrinone

Supplemental additional data Table S2

Conversion from Venovenous to Venoarterial Extracorporeal Membrane Oxygenation in Adults

Lars Falk[1,2], Alexander Fletcher-Sandersjöö[3,4], Jan Hultman[1,2], Lars Mikael Broman[1,2]

1) ECMO Centre Karolinska, Department of Pediatric Perioperative Medicine and intensive Care, Karolinska University Hospital, Stockholm, Sweden; 2) Department of Physiology and Pharmacology, Karolinska Institutet, Stockholm, Sweden; 3) Department of Clinical Neuroscience, Karolinska Institutet, Stockholm, Sweden; 4) Department of Neurosurgery, Karolinska University Hospital, Stockholm, Sweden

Table S2: Data and outcome after conversion from venovenous to venoarterial extracorporeal membrane oxygenation

Variables and findings at time of conversion from venovenous to venoarterial ECMO in 46 patients. The four patients marked in italic were not included in the detailed analyses of RVF and LVF. The indications for conversion in these subjects were unknown

Pat no.	pH	SaO2 SpO2 %	Lact mmol/L	FiO2 %	PIP cmH2O	PEEP cmH2O	Vt mL	MAP mmHg	SAVE	SOFAconv	SOFA delta	SOFA circ	ECHO w. RVF	No. vasoactive drugs	VIS	Death on ECMO
**1**	7.52	66	0.8	4 L/min oxygen				56	-8	10	1	3	yes	1	6	
**2**	7.29	97	1.1	40	28	5	68	73	-6	12	0	4	yes	2	10	yes
**3**	7.32	53	4.8	60	22	5	278	51	-7	15	4	4	yes	1	14	yes
**4**	7.24	89	0.9	50	25	1	242	45	-10	13	6	4	yes	3	15	yes
**5**	7.59	77	1.1	50	24	2	18	69	-4	11	2	3	yes	1	1.1	yes
**6**	7.53	87	1.1	100	25	4	330	53	-9	11	-2	0	yes	0	0	yes
**7**	7.42	94	2.0	60	15	0	320	76	-10	16	3	4	yes	2	14.5	yes
**8**	7.43	78	1.2	40	21	5	8	66	-6	8	0	0	yes	0	0	
**9**	7.41	70	1.4	50	25	4	73	67	-7	14	4	4	yes	3	12	
**10**	7.27	90	1.1	50	16	3	260	75	-10	13	3	3	yes	2	9	
**11**	7.40	72	1.3	50	20	1	70	79	-5	14	6	0	yes	1	2	yes
**12**	7.45	76	1.7	80	25	9	114	73	-10	14	3	4	yes	1	10	yes
**13**	7.39	82	0.7	50	23	10	405	61	-7	12	0	3	yes	2	8	yes
**14**	7.31	89	2.0	60	34	14	309	66	-10	16	0	4	no	3	34	
**15**	7.43	71	1.1	60	21	6	45	77	-3	9	1	0	no	1	Isopr	yes
**16**	7.31	73	1.6	50	12	1	41	64	-3	13	4	4	yes	2	83	
**17**	7.31	80	2.0	60	26	5	86	59	-6	15	9	4	yes	1	32	yes
**18**	7.25	58	0.8	100	25	0	100	81	-1	12	1	4	yes	2	28.5	
**19**	7.41	70	1.4	70	18	1	184	65	-4	17	9	4	yes	1	17	
**20**	7.28	59	6.3	60	26	8	100	64	-5	15	1	4	no	3	24	
21	*7.28*	*72*	*7.6*	*50*	*20*	*10*	*20*	*58*	*-9*	*15*	*1*	*4*	*yes*	*2*	*21*	
22	*7.31*	*95*	*4.5*	*50*	*28*	*8*	*545*	*70*	*-12*	*14*	*1*	*4*	*yes*	*2*	*52*	*yes*
**23**	7.36	68	1.6	50	24	3	54	75	-13	16	4	3	yes	1	9	yes
**24**	7.40	97	7.1	90	23	5	200	54	-4	17	4	4	no	3	61	
**25**	7.35	72	1.2	50	23	9	49	70	-9	14	6	4	yes	2	21	yes
**26**	7.27	87	1.8	100	32	12	418	90	-13	15	3	4	no	1	24	
**27**	7.41	64	1.7	40	22	6	37	64	-8	15	6	3	yes	1	1	yes
**28**	7.39	83	2.3	40	23	5	47	71	-1	13	1	0	no	0	0	
**29**	7.38	62	2.9	60	30	8	134	55	-10	16	0	4	yes	2	35	yes
**30**	7.32	61	6.2	80	33	7	117	90	-16	17	12	4	yes	1	15	yes
**31**	7.35	92	8.6	60	27	10	234	68	-20	20	7	4	no	3	92	yes
**32**	7.37	81	3.3	70	25	12	391	69	-5	14	6	4	no	1	27	
**33**	7.44	90	1.7	No ventilation				66	-4	19	5	3	yes	1	5	yes
**34**	7.43	87	3.2	60	29	5	122	84	-19	17	3	3	yes	2	16	yes
**35**	7.36	82	1.9	60	26	9	419	69	-11	17	5	3	yes	2	15	

**36**	7.37	76	2.1	80	34	6	200	71	-7	14	3	4	yes	1	14	yes
**37**	7.42	96	0.7	50	21	5	63	71	-7	14	1	3	yes	1	2	yes
**38**	7.38	77	1.5	100	28	8	317	63	-11	17	3	4	yes	1	20	yes
**39**	7.26	90	14.2	60	26	7	357	54	-12	19	5	4	no	2	96	yes
**40**	7.40	81	4.2	50	20	6	670	55	-14	17	10	4	yes	2	27	yes
**41**	7.37	82	1.3	50	25	7	134	55	-11	18	2	4	yes	1	13	
42	*7.38*	*82*	*1.2*	*80*	*22*	*8*	*165*	*70*	*-12*	*14*	*5*	*4*	*no*	*1*	*27*	*yes*
**43**	7.29	77	6.8	50	25	15	42	64	-7	18	2	4	no	3	24	yes
**44**	7.39	86	1.4	60	27	7	64	72	-8	12	0	4	yes	2	30	
45	*7.39*	*89*	*2.8*	*60*	*25*	*10*	*298*	*62*	*-6*	*17*	*5*	*4*	*no*	*2*	*22*	
**46**	7.32	71	3.8	80	28	9	128	63	-10	18	2	4	no	2	38	yes

Abbreviations: Lact, plasma lactate; FiO2, fraction inspired oxygen; PIP, peak inspiratory pessure; PEEP, positive end-exppiratory pressure; Vt, tidal volume; MAP, mean arterial blood pressure; Hgb, hemoglobin concentration; SAVE, Survival After Venoarterial ECMO score; SOFA conv, Sequential Organ Failure Assessment score at time of conversion; SOFA delta, difference between SOFA score at admission and SOFA score at conversion (positivive digit indicates worsening of organ failures, and *vice versa* ; SOFA-c, SOFA score for circulatory domain; ECHO, echocardiography; VIS, vasoactive inotropic score
